# Biocompatibility of New Hydrogels Based on a Copolymer of Fish Collagen and Methyl Methacrylate Obtained Using Heterogeneous Photocatalysis Under the Influence of Visible Light

**DOI:** 10.3390/polym17152002

**Published:** 2025-07-22

**Authors:** Victoria Rumyantseva, Lyudmila Semenycheva, Natalia Valetova, Marfa Egorikhina, Ekaterina Farafontova, Daria Linkova, Ekaterina Levicheva, Diana Fukina, Evgeny Suleimanov

**Affiliations:** 1 The Research Institute for Chemistry, Lobachevsky State University of Nizhny Novgorod, pr. Gagarina 23, 603950 Nizhny Novgorod, Russia; llsem@yandex.ru (L.S.); nata-bor-2005@mail.ru (N.V.); dianafuk@yandex.ru (D.F.); suev@unn.ru (E.S.); 2 Federal State Budgetary Educational Institution of Higher Education, Privolzhsky Research Medical University, The Ministry of Health of the Russian Federation (FSBEI HE PRMU MOH), Minin and Pozharsky, Square 10/1, 603005 Nizhny Novgorod, Russia; egorihina.marfa@yandex.ru (M.E.); ekaterina_farafontova@mail.ru (E.F.); linckovadaria@yandex.ru (D.L.); kate.lekat@yandex.ru (E.L.)

**Keywords:** new hydrogel, cod collagen, methyl methacrylate, polyethylene glycol, RbTe_1.5_W_0.5_O_6_ complex oxide, MTT assay, tissue engineering

## Abstract

New stable three-dimensional hydrogels were obtained in an inert gas atmosphere in light in an aqueous dispersion of the main components: cod collagen, methyl methacrylate, polyethylene glycol, RbTe_1.5_W_0.5_O_6_ complex oxide, and modifying additives. The analysis of the new hydrogels’ cytotoxicity using the MTT assay showed that the cytotoxicity of the sample extracts was observed in a number of examples, but was decreased with increasing dilution of the extracts. The decrease in cell viability at high concentrations of the extract is likely caused by a decrease in the number of specific components of the complete culture medium used to produce extracts. It is related to the well-known adsorption of medium proteins by the gel component, high-molecular compounds included in the matrix. The stimulating effect of the substances included in its composition was observed with a significant dilution of the extract, i.e., the proliferative activity of the cells increased. The extract of the hydrogel hydrolysate sample and all its dilutions did not show cytotoxicity in the MTT assay examples. It determines the prospect of its use on the wound surface, since hydrogel destruction occurs under the action of body enzymes. The new hydrogel is a promising material for creating wound coverings or scaffolds.

## 1. Introduction

Natural polymer-based materials are used in various wound-healing formulations of any etiology. Its development is one of the most widely used approaches in tissue engineering, as it can be used to create a three-dimensional structure that can support growth and differentiation of cells. Well-known available natural polymer materials, especially collagen, have been extensively studied in this regard and are presented in the literature. Research has shown that collagen has various biological properties, including antioxidant, osteogenic differentiation, and antihypertensive and antidiabetic properties [1,2,3,4]. Collagen hydrogels are used in dressings, as drug carriers in medicine, and in tissue engineering. The main reason for the need to model natural polymers in wound-healing coatings is the lack of a stable three-dimensional structure [5,6,7,8,9,10]. This problem is increasingly being solved by adding modifying additives, as well as obtaining hybrid materials with the inclusion of synthetic fragments that give the material the desired properties [11,12,13,14,15]. Fish collagen has become the subject of study in the field of medical materials due to its biocompatibility and valuable bioactive components, the exclusion of the possibility of disease transmission from animals to humans, and the absence of religious and ethical restrictions in comparison with its animal equivalent [6,16,17,18,19,20]. At the same time, the use of catalytic processes in the framework of “green chemistry”, in particular, heterogeneous photocatalysis under mild irradiation (daylight, LED lamps, etc.), has become an innovative strategy in chemistry, including in the synthesis of new polymer materials. For example, methyl methacrylate (MMA) is an important monomer for the production of not only acrylic plastics and polymer dispersions, but also as a promising additive for oxygenated fuels to increase combustion efficiency and reduce emissions [21], but also new materials in medicine [22,23,24]. The most well-known research in the synthesis of polymeric materials is on the use of photocatalysis in controlled radical polymerization, when the control of radical polymerization is an important problem for macromolecular engineering. In this case, photocatalysis has found applications for the regeneration of chain breakage control agents that react with growing polymer chains to form “sleeping species” [25,26]. The authors [27,28,29,30,31,32,33,34,35,36,37] obtained hydrogels of a stable three-dimensional structure with a wide pore range from several nm to 70 nm in an inert atmosphere and visible light photocatalysis conditions in the presence of RbTe_1.5_W_0.5_O_6_ oxide in an aqueous dispersion of cod collagen (CC) and MMA with modifiers. The hydrogel easily releases water when dried in a vacuum. Its undoubted advantage is its biocidal effect on fungi. From the point of view of the structure and nature of the components included in the hydrogel, it is promising as a cellular matrix carrier or drug delivery system. It is well known that the components of the material: collagen, polyethylene glycol (PEG), and polyacrylates have long been used in medical products [38,39]. The continued research of new hydrogels is related to the biocompatibility of the material. The aim of this work is to analyze the cytotoxicity of new hydrogels based on the CC-PMMA copolymer using an MTT assay, which will assess the metabolic activity of cells and assess their viability. To assess possible manifestations of the toxicity of hydrogel fragments, MTT assay analysis was performed for hydrogels obtained under conditions of photocatalysis in an aqueous dispersion of the main components as follows:

- cod collagen, methyl methacrylate, polyethylene glycol, acrylic acid (AA), complex oxide RbTe_1.5_W_0.5_O_6_, and small amounts of modifying additives (triethylene glycol dimethacrylate (TEGDMA))—a sample of CCC, described in the work [34];
- cod collagen, methyl methacrylate, polyethylene glycol, AA, complex oxide RbTe_1.5_W_0.5_O_6_, and small amounts of modifying additives (TEGDMA, glutaraldehyde (GA))—sample CCC-G, described in the work [39];
- the product of enzymatic destruction of the CCC sample is CCC–H hydrolysate.

Collagen-based hydrogels represent the most important class of biomaterials due to their desired physicochemical and biochemical properties. Changes in the composition, gelation conditions, and crosslinking methods can affect the physicochemical and biological properties of collagen-based hydrogels. However, the specific effect of these parameters on the mechanisms of gelation of new hydrogels and the relationship between the production parameters and the characteristics of these hydrogels remain unclear.

## 2. Materials and Methods

### 2.1. Materials

Commercial reagents were used: acetic acid (analytical grade, Lega, Dzerzhinsk, Russia), sodium hydroxide (pure for analysis, Reahim, Moscow, Russia), proteolytic enzyme pancreatin (proteolytic activity in 1 mL—2IU), chloroform (analytical grade, Baza №1 Himreaktivov, Staraya Kupavna, Russia), acrylic acid (pure for analysis, Sigma Aldrich, Burlington, MA, USA), triethylene glycol dimethacrylate (Chemtransite, Dzerzhinsk, Russia), and polyethylene glycol (Mw = 6000, Norkem, Dzerzhinsk, Russia). A monomer, methyl methacrylate (pure for analysis, Sigma Aldrich, St. Louis, MO, USA) was used. It was purified from the stabilizer by sequential washing with a solution of sodium hydroxide and cold water until a neutral pH was reached. It was then dried using calcium chloride and distilled in a vacuum (1.33 Pa) at a temperature of 40 °C. The complex oxide RbTe_1.5_W_0.5_O_6_ was obtained by the solid-state method, as described earlier [40].

### 2.2. Isolation of Cod Collagen

Collagen was isolated according to the method described in [41] by extraction with acetic acid for one day at room temperature. The resulting acetic acid dispersion was dried to a constant weight under a vacuum (1.33 Pa) at 50 °C.

### 2.3. Synthesis of CCC and CCC-G Copolymer

The CCC sample was synthesized according to the previously described method [34]. CC, MMA, TEGDMA, AA, PEG, and water were mixed in a reaction flask (7.7:3.80:0.05:3.80:7.7:76.88 wt.% accordingly), a complex oxide RbTe_1.5_W_0.5_O_6_ was added in the emulsion:catalyst ratio = 180:1. The mixture was bubbled with argon for 15 min, then stirred (600 rpm) under irradiation with a visible-light-emitting diode lamp (LED, 30 W, IEK, Moscow, Russia) in argon current for 5 h. Then the catalyst was separated by centrifugation and the solution was freeze-dried. Part of the sample was examined in the form of a lyophilized sponge—CCC; part of the sample was additionally extracted with chloroform and dried again to a constant weight—CCC-Ch. The CCC-G sample was obtained by introducing an aqueous solution of glutaraldehyde into the initial reaction mixture in an argon current and keeping it at room temperature. The resulting crosslinked CCC-G copolymer was dried in a vacuum (1.33 Pa) to a constant weight at 50 °C.

### 2.4. Enzymatic Hydrolysis of the CCC Sample

Hydrolysis by the proteolytic enzyme pancreatin was performed at room temperature at pH 7.0 (CC:pancreatin ratio = 10^3^:1). To stop the reaction after 3 days, a 4% solution of acetic acid was added to the reaction mixture. Next, the resulting hydrolysate was dried in a vacuum (1.33 Pa) to a constant weight at 50 °C—sample CCC-H.

### 2.5. Cytotoxicity Assessment—MTT Assay

In order to assess the hydrogel samples’ cytotoxicity, an MTT assay was performed in accordance with the recommendations [42]. To obtain the extract, the test samples were placed in a Dulbecco’s Modified Eagle Medium (DMEM)/F12 growth medium with the addition of antibiotics and 2% fetal bovine serum (FBS) and incubated in a CO_2_ incubator (Shellab 3517-2; Sheldon Manufacturing, Cornelius, OR, USA) for 24 h. After obtaining the extracts, they were sterilized by filtration through filters of 0.22 microns. Simultaneously with the start of extraction, the cells of the test culture, human dermal fibroblasts (HDFs), were seeded with a cell concentration of 100 thousand cells/mL per well of a 96-well tablet in a full growth medium (DMEM/F12 medium with the addition of antibiotics penicillin/streptomycin, glutamine, and 10% FBS) and cultured in a CO_2_ incubator for 24 h. All reagents and media used, unless specified separately, were from PanEco, Moscow, Russia. The cultures were obtained and characterized in the Laboratory of Biotechnology of the FSBEI HE PRMU of the Ministry of Health of the Russian Federation. Obtaining and using biological material for research was approved by the local Ethics Committee of the Russian Ministry of Health on 30 June 2023, Protocol No. 9. The method of obtaining cultures was described in detail by us earlier [43]. The obtained extracts were diluted with growth medium in the ratio: 1:1; 1:2; 1:4; 1:8. After that, the extracts and their dilutions were poured into the prepared test culture in a flat-bottomed 96-well plate, with 8 holes of the tablet each. After 72 h of cultivation with extracts, the state of the culture on the surface of the experimental and control wells was assessed using an inverted microscope Leica DMI 3000 B (Leica Microsystems, Wetzlar, Germany, software LAS v.4.3). Then, 20 µL of MTT solution was added to each well and placed in a CO_2_ incubator for 3 h. After that, the supernatant was selected, replaced with an equal volume of dimethyl sulfoxide (DMSO) solution, and the optical density was recorded at 540 nm on an analyzer INFINITI F50, Tecan photometer (Tecan Austria GmbH, Grödig, Austria) with Magellan software V7.2 (Tecan Austria, Grödig, Austria). The cytotoxicity of the daily extract and its dilutions in comparison with control samples was assessed by the relative intensity of cell growth in the test culture (RGR).


$$\mathrm{RGR}\left(\%\right)=\frac{the\;average\;OD\;in\;the\;experimental\;series}{average\;OD\;in\;control}\times 100$$

RGR—relative growth rate, OD—optical density.

To assess cytotoxicity, the following ranks were identified: rank 0 (RGR = 100%) and rank 1 (RGR = 99–70%) correspond to the absence of cytotoxicity, rank 2 (RGR = 69–50%) corresponds to mild cytotoxicity, rank 3 (RGR = 49–25%) corresponds to moderate degree, and rank 4–5 (RGR = 24–0%)—marked cytotoxicity.

### 2.6. Statistical Analysis

Statistical analysis was performed using the STATISTICA 6.0 (Dell Technologies Inc., Round Rock, TX, USA) software system. This study was conducted using methods of nonparametric statistics and the Wilcoxon paired comparison test.

## 3. Results and Discussion

**Figure 1:**
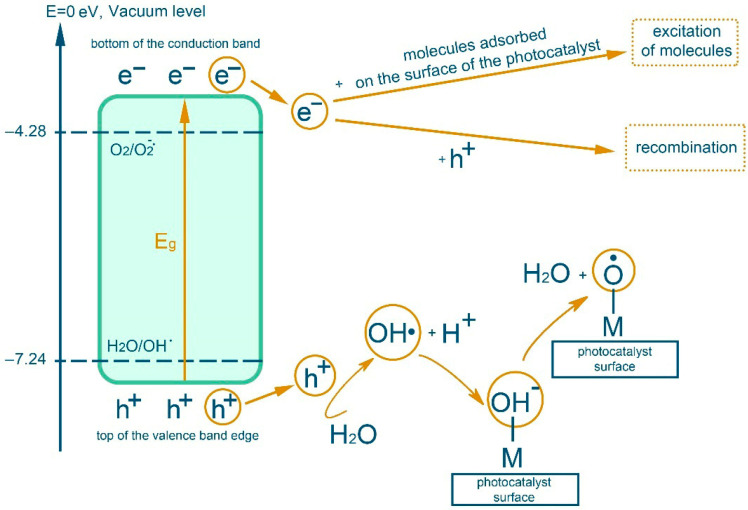
Scheme of transformations during photocatalysis in aqueous dispersion with complex oxides in an inert gas atmosphere.

In an inert gas atmosphere, redox processes take place in an aqueous dispersion component of polymerizate and RbTe_1.5_W_0.5_O_6_ complex oxide, which lead to a number of transformations according to the scheme in Figure 1 [27,35].

**Figure 2:**
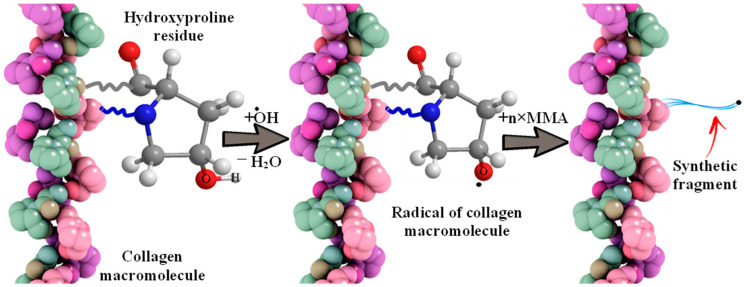
The scheme of interaction of a hydroxyl radical with a collagen molecule and MMA grafting.

The interaction of the hydroxyl radical with the organic substrates of the reaction mixture leads to the formation of stable three-dimensional structures, hydrogels, described earlier [27,28,29,30,31,32,33,34,35,36]. Namely, due to the separation of a hydrogen atom by a hydroxyl radical formed by irradiation with visible light (λ = 400–700 nm) of the complex oxide RbTe_1.5_W_0.5_O_6_ from collagen macromolecules (from the hydroxyl group of hydroxyproline or from the hydrocarbon part of amino acid residues), which will lead to the formation of oxygen- or of a carbon-centered radical, respectively. In the presence of a monomer, MMA is grafted onto fish collagen (Figure 2 using hydroxyproline as an example).

**Figure 3:**
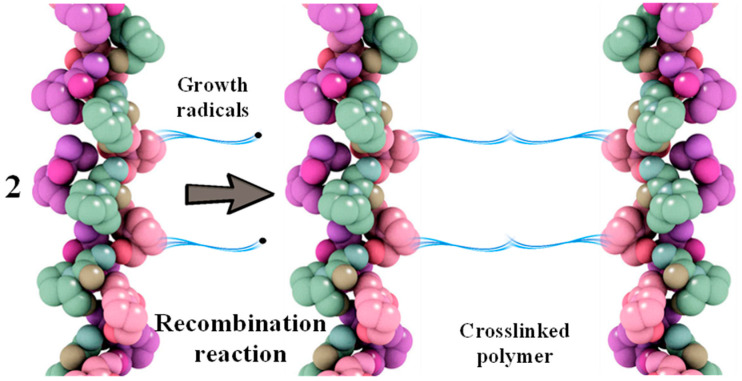
Scheme of recombination reaction of grafted polymer radicals based on collagen and MMA.

Recombination reactions of growing macroradicals lead to crosslinking between protein macromolecules (Figure 3) [44].

**Figure 4:**
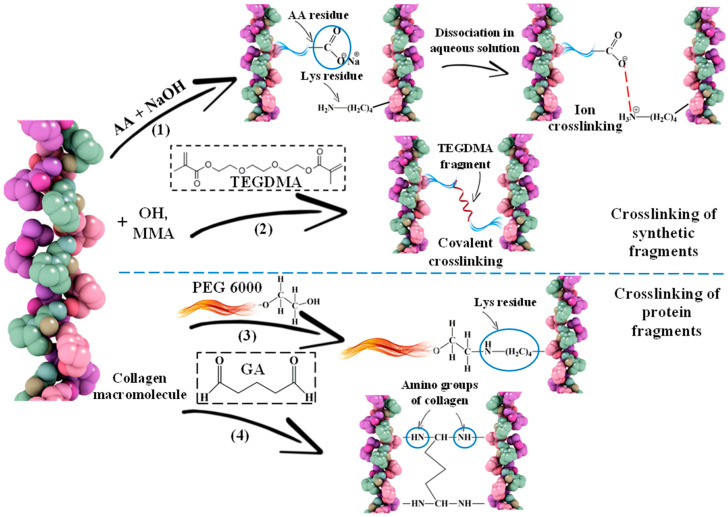
Schemes of crosslinking of collagen macromolecules due to synthetic (**1**,**2**) and protein (**3**,**4**) fragments: (**1**) adding AA to the system and neutralizing the mixture; (**2**) adding TEGDMA; (**3**) adding PEG; (**4**) adding GA.

In addition, the additional crosslinking described earlier in the work [34,39], is formed by the introduction of AA, TEGDMA (Figure 4(1,2)) and PEG, GA (Figure 4(3,4)).

**Figure 5:**
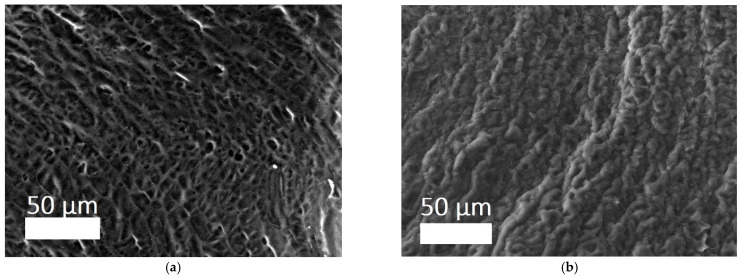
SEM images of sponges of hydrogels: (**a**) CCC; (**b**) CCC-G [39].

The 3D structure of the hydrogels was determined using a scanning electron microscope (SEM) (Figure 5a,b). It can be seen that the filamentous extended collagen macromolecules are crosslinked, forming a porous framework with pores from several microns to ~70 microns. The porous structure of hydrogels allows one to imitate the structure of native tissue environment, making the exchange of nutrients and the formation of cellular connections easier, which are processes of critical importance for tissue regeneration [45,46]. Moreover, the possibilities of cell migration and vascularization are related to the size and presence of interpenetrating pores.

Along with this, data on the structural stability of hydrogels at a specific pH (6.8–7.4) have been obtained, which helps optimize the microenvironment of the wound bed and maintain regenerative processes. There is also evidence of good moisture absorption: the dried gel quickly, within a few minutes, absorbs a mass of water several times greater than its own weight of the hydrogel. This property is able to ensure the absorption of exudate in the wound when using the matrix as a wound coating [39].

The next stage in the development of a material for use in regenerative medicine is the combination of hydrogels with stem cells. This problem is solved primarily by biological analysis of biocompatibility, using the MTT assay in this work.

**Figure 6:**
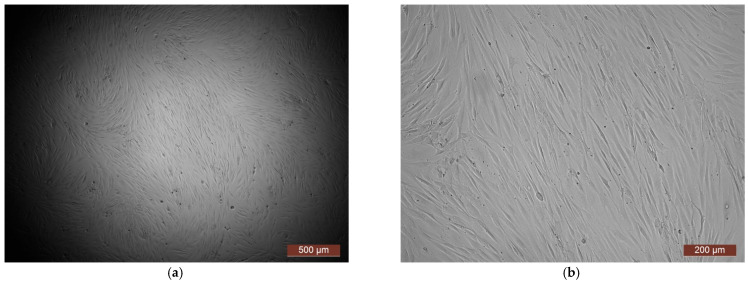
Representative photos of the test culture (human dermal fibroblasts) before contact with the samples (24 h of cultivation): (**a**) magnification of 40×; (**b**) magnification of 100×.

**Figure 7:**
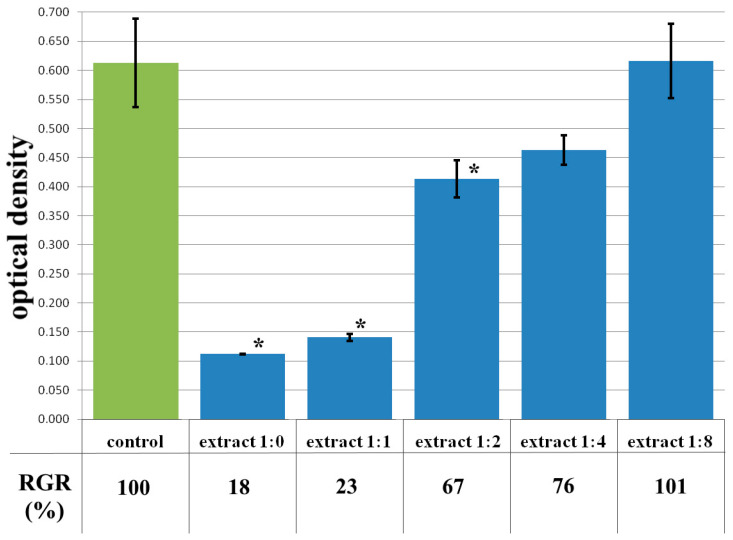
Optical density in the analysis of the extract of the CCC sample and its dilutions: green color—control sample of HDFs without the CCC copolymer; blue color—sample of HDFs with extract of the CCC sample and its dilutions. Note: * *p* < 0.05—comparison with the control, Wilcoxon criterion.

**Figure 8:**
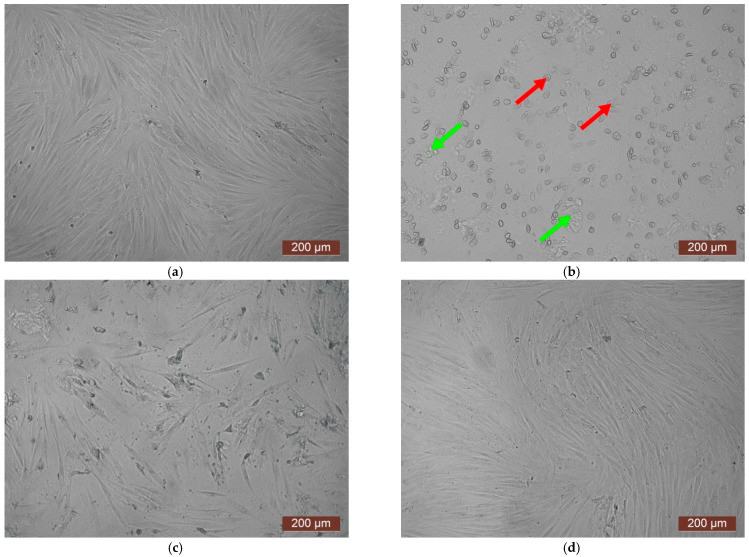
Representative photos of the cell culture state after cultivation with the extract of the CCC sample (24 h of cultivation, magnification 100×): (**a**) control; (**b**) extract 1:1, red arrows—spherical HDF, green arrows—fragments of the sample; (**c**) extract 1:2; (**d**) extract 1:4.

The cytotoxicity of the samples was assessed using an MTT assay and using human dermal fibroblasts as test cultures. Before the introduction of cell cultures into the experiment, a subconfluent monolayer was formed, the cells had a characteristic fusiform shape with pronounced appendages (Figure 6). The cytotoxicity studies of the CCC sample showed that the daily extract and its 1:1 dilution showed marked cytotoxicity—rank 4 (Figure 7). The microscopic picture corresponded to the obtained colorimetric analysis data. Almost all the cells in the visual field had a spherical shape, which confirmed the toxic effect of the extract on the cells (Figure 8b). It is widely known that surface-dependent cells can take on a spherical shape and detach from the surface under unfavorable conditions. Such a morphological change usually occurs when the components of the solution are toxic to the cell culture, disrupting the adhesion of surface-dependent cells to the culture plastic [47]. It should be noted that a slight decrease in cell viability at high concentrations of the extract might be due not to the release of cytotoxic substances from the gel, but to a decrease in the number of specific components of the complete culture medium used to produce extracts. It is related to the known adsorption of medium proteins by the gel component, high-molecular compounds that make up the matrix: CC, PMMA, PEG. This fact is well known and discussed in the literature [48,49,50]. During microscopic study, relief translucent structures were visualized in the wells with the extract, apparently separated particles of the sample. When analyzing the extract of the test sample at a dilution of 1:2, it was found that the cytotoxic effect was lower than that of the whole extract and its dilution of 1:1 and corresponded to rank 2 (Figure 7). The picture of the cells’ state during microscopy corresponded to a mild degree of cytotoxicity. Both spherical cells and those spread over the surface of the culture plastic with typical morphology were visualized (Figure 8c). With further dilution of the extract, the cytotoxic effect was leveled, and the results of the MTT assay for dilutions 1:4 and 1:8 corresponded to rank 1–0 (Figure 7). The microscopic picture of dilutions 1:4 and 1:8 practically did not differ from the control (Figure 8d). Thus, it is possible to talk about a decrease in the toxic effect two ways. On the one hand, it could be observed with a decrease in the concentration of substances released from the sample, causing a cytotoxic effect. On the other hand, it could be related to a decrease in the number of specific components of the complete culture medium used to produce extracts, due to the known adsorption of proteins of the medium by the gel component.

**Figure 9:**
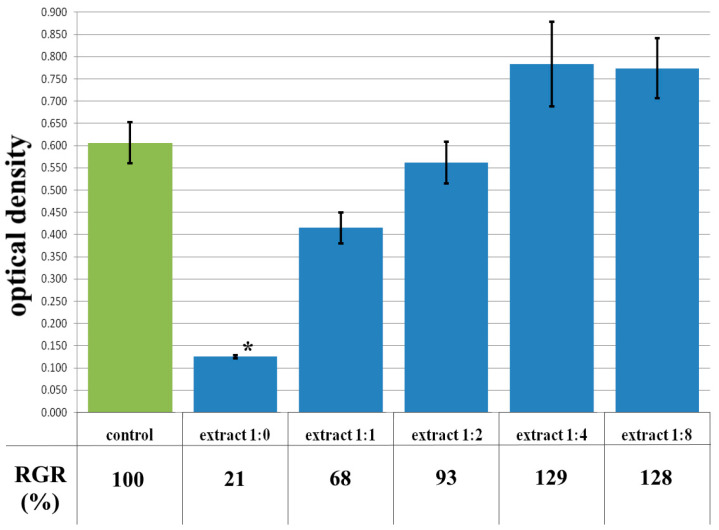
Optical density in the analysis of the extract of the CCC-Ch sample and its dilutions: green color—control sample of HDFs without the CCC-Ch copolymer; blue color—sample of HDFs with extract of the CCC-Ch sample and its dilutions. Note: * *p* < 0.05—comparison with the control, Wilcoxon criterion.

**Figure 10:**
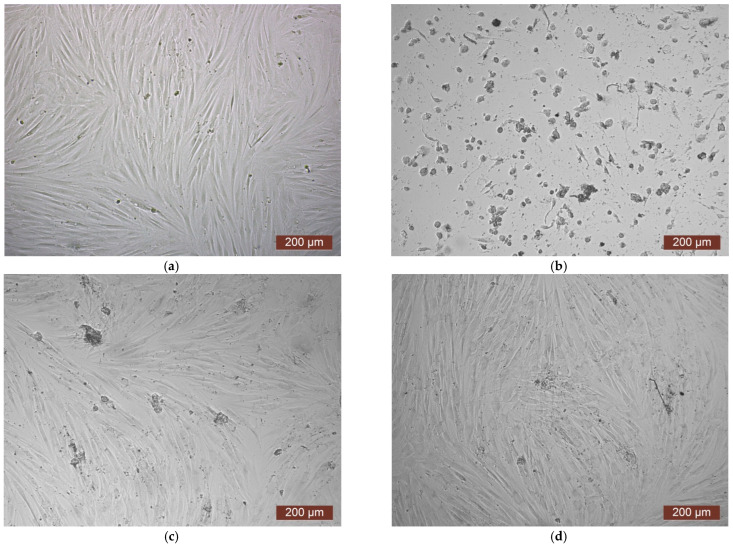
Representative photos of the cell culture state after cultivation with the extract of the CCC-Ch sample (24 h of cultivation, magnification 100×): (**a**) control; (**b**) extract 1:0; (**c**) extract 1:1; (**d**) extract 1:2.

The MTT assay of the extract of the CCC-Ch sample also showed toxicity, which corresponded to rank 4 cytotoxicity (Figure 9). The visual picture reflected the results of colorimetric analysis—almost all cells in the extract either had a spherical shape or were deformed (Figure 10b). A 1:1 dilution study of the extract showed a mild degree of toxicity corresponding to rank 2 (Figure 9). The reasons for this reaction were discussed in the article earlier. The microscopic picture confirmed a decrease in cytotoxicity—in a 1:1 dilution series of the extract, a subconfluent cell monolayer was visualized, as well as a small amount of cellular detritus and single spherical cells (Figure 10c). Dilutions of the extract 1:2–1:8 demonstrated the absence of cytotoxicity—rank 1–0 (Figure 9). The microscopic picture of these dilutions of the extract was a confluent cell monolayer, visually identical to the control (Figure 10d).

It should be noted that with a significant dilution of the extract (1:4 and 1:8), the OD exceeded the control values, respectively; the RGR was also higher than 100% (129% for dilution of extract 1:4; 127% for dilution of extract 1:8) (Figure 9). An increase in the proliferative activity of the cells is indicated, and, consequently, a certain stimulating effect of the substances included in the extract in low concentrations. However, given the absence of statistically significant differences in relation to the control, we can only speak of a tendency to stimulate cell growth.

Thus, when comparing samples of CCC and CCC-Ch according to the results of the MTT assay, similar values of the parameters were observed. It indicates the absence of toxic inclusions in the hydrogel after freeze-drying, i.e., the stage of extraction of organic toxic fragments with chloroform can be excluded from the “chain” of hydrogel preparation for biocompatibility tests.

**Figure 11:**
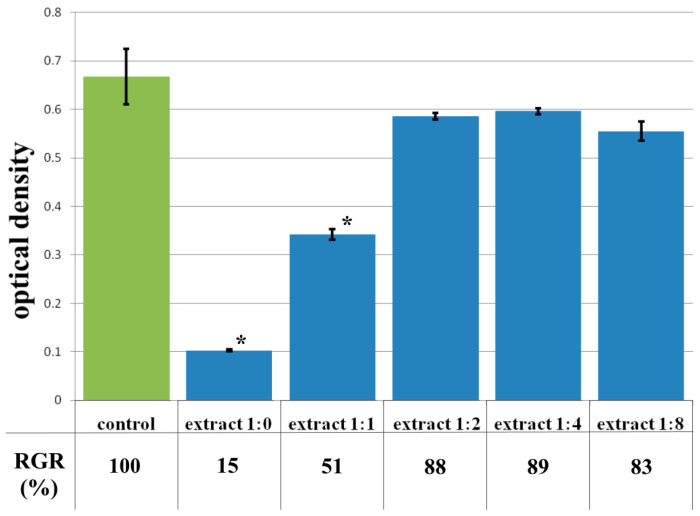
Optical density in the analysis of the extract of the CCC-G sample and its dilutions: green color—control sample of HDFs without the CCC-G copolymer; blue color—sample of HDFs with extract of the CCC-G sample and its dilutions. Note: * *p* < 0.05—comparison with the control, Wilcoxon criterion.

**Figure 12:**
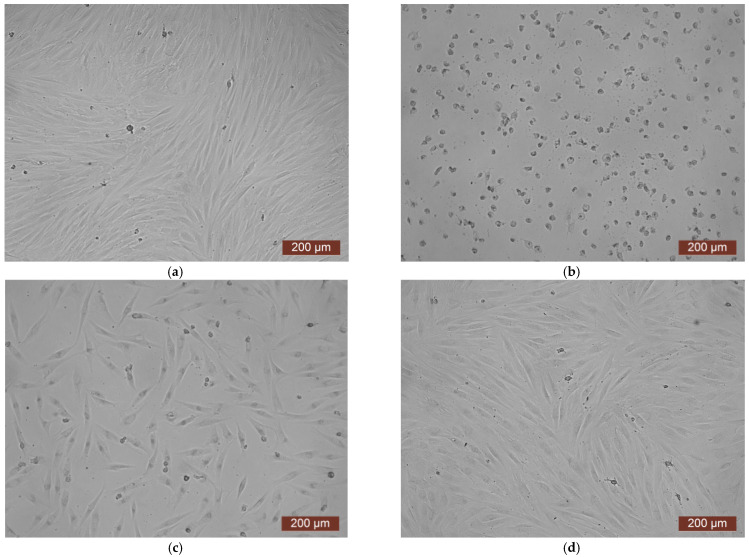
Representative photographs of the cell culture state after cultivation with the extract of the CCC-G sample (24 h of cultivation, magnification 100×): (**a**) control; (**b**) extract 1:0; (**c**) extract 1:1; (**d**) extract 1:2.

The data from the cytotoxicity study of the CCC-G sample demonstrated that the daily extract showed rank 4 toxicity (Figure 11). Spherical cells were mainly fixed in wells with the whole extract of the sample (Figure 12b). The study of the dilution of the extract 1:1 showed that the OD corresponded to the 2nd rank of cytotoxicity (Figure 11). The visual picture was similar: the spread cells were mainly spindle-shaped, forming intercellular contacts with the formation of a cellular network (Figure 12c). The explanation for this fact has already been discussed earlier. With further dilution of the extract, the cytotoxic effect was leveled, and the results of the MTT assay for dilutions of 1:2–1:8 corresponded to rank 1 (absence of cytotoxicity) (Figure 11). The microscopic picture in the study of dilutions of the extract of 1:2–1:8 practically did not differ from the control: the cell culture formed a confluent monolayer formed by typical elongated fibroblasts, spindle-shaped, with pronounced appendages (Figure 12d).

**Figure 13:**
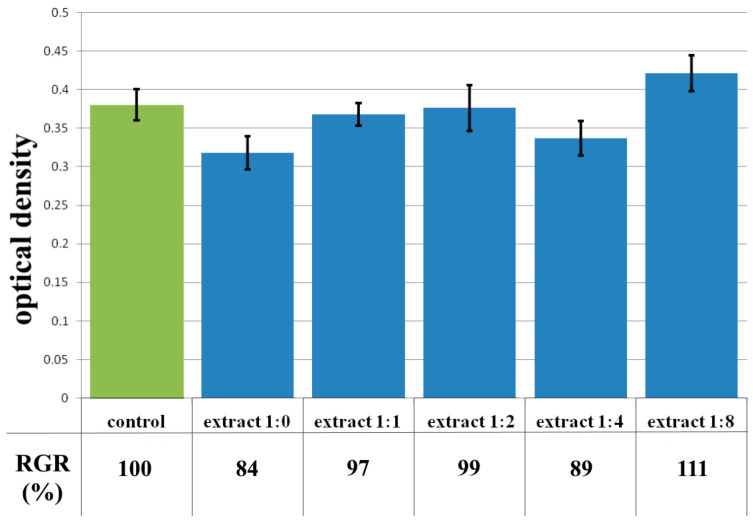
Optical density in the analysis of the extract of the CCC-H sample and its dilutions: green color—control sample of HDFs without the CCC-H copolymer; blue color—sample of HDFs with extract of the CCC-H sample and its dilutions.

**Figure 14:**
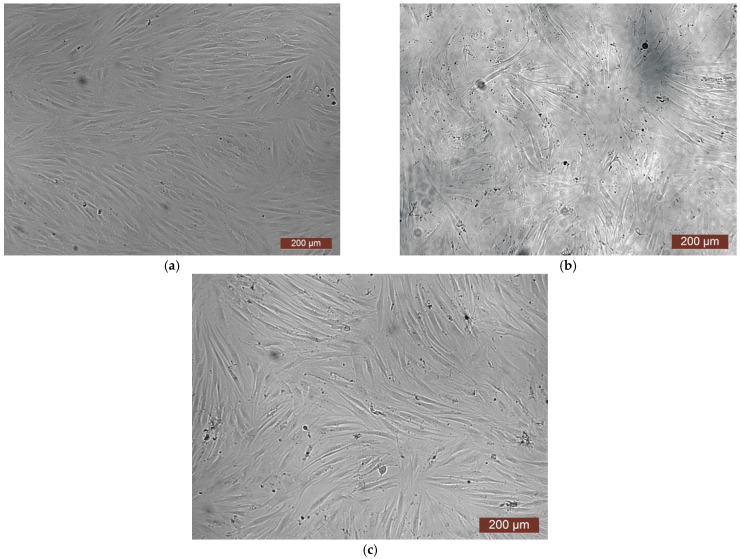
Representative photos of the cell culture state after cultivation with the extract of the CCC-H sample (24 h of cultivation, magnification 100×): (**a**) control; (**b**) extract 1:0; (**c**) extract 1:1.

The extract of the CCC-H sample and its dilutions, unlike previous samples, did not show cytotoxicity. Thus, colorimetric analysis showed that the OD of both the extract and its dilutions of 1:1–1:8 corresponded to the rank 1–0 of cytotoxicity (Figure 13). During microscopic examination, the culture of HDF under the action of the extract in dilutions of 1:1–1:8 represented a confluent monolayer formed by spindle-shaped cells tightly adjacent to each other, which corresponded to the control (Figure 14c). Visually, it was possible to note the difference between the condition of the cells of the test culture in the whole extract series and the condition of the cells in the control series. In the whole extract series, the cells of the test culture retained their typical morphology, but the visual density of cells on the surface was reduced (Figure 14b).

The absence of toxicity of CCC-H hydrolysate are important in the sense that, as is known [51,52], the destruction of the hydrogel in case of its use on the wound surface occurs under the action of enzymes. In this case, we observe the absence of release of toxic hydrogel fragments.

## 4. Conclusions

The study of the cytotoxicity of new CC-PMMA-based hydrogels using the MTT assay was shown that the cytotoxicity of sample extracts observed in a number of examples and is leveled with increasing dilution of extracts. In addition, a decrease in cell viability at high concentrations of the extract may be due not to the release of cytotoxic substances from the gel, but to a decrease in the number of specific components of the complete culture medium used to produce extracts. This is related to the known adsorption of media proteins by the gel component, high-molecular compounds that make up the matrix. Dilution of the extract led to a stimulating effect of the substances included in its composition, i.e., increased cell proliferative activity. The MTT assay data from the hydrogel hydrolysate sample extract and all its dilutions did not show cytotoxicity. The latter allows us to assume that the destruction of the hydrogel in the case of its use on the wound surface, which occurs under the action of enzymes, does not lead to the release of toxic fragments of the hydrogel. Thus, the proposed new hydrogel is a promising material for creating hydrogel wound coatings. Due to the previously mentioned properties of collagen, such as antioxidant, osteogenic differentiation, etc., such wound coatings will accelerate wound healing. The prospect of using new hydrogels in scaffold technologies is related to the properties of collagen and the MTT assay data on cell viability in gel extracts obtained in this work.

## Data Availability

The original contributions presented in this study are included in the article. Further inquiries can be directed to the corresponding author.
